# Navigator-gated free-breathing joint T_1_–T_2_ mapping of the kidney

**DOI:** 10.1007/s10334-026-01337-8

**Published:** 2026-03-05

**Authors:** Pauline Calarnou, Augustin C. Ogier, Christopher W. Roy, Jean-Baptiste Ledoux, Angela Rocca, Stanislas Rapacchi, Menno Pruijm, Roger Hullin, Jean-Paul Vallée, Jérôme Yerly, Ruud B. van Heeswijk

**Affiliations:** 1 Department of Radiology, Lausanne University Hospital and University of Lausanne, CHUV BH, 08/084, Rue du Bugnon 46, 1011 Lausanne, Switzerland; 2 CIBM Center for BioMedical Imaging, Lausanne, Geneva, Switzerland; 3 Department of Cardiology Service, Cardiovascular, Lausanne University Hospital and University of Lausanne, Lausanne, Switzerland; 4 Department of Nephrology Service, Internal Medicine, Lausanne University Hospital and University of Lausanne, Lausanne, Switzerland; 5 Radiology Service, Department of Diagnostics, Geneva University Hospital and University of Geneva, Geneva, Switzerland

**Keywords:** Magnetic resonance imaging, Kidney, Parametric mapping, Free breathing, Dictionary fitting

## Abstract

**Objective:**

To develop and evaluate a free-breathing 2D radial joint T₁–T₂ mapping technique for the kidneys at 3T, and to assess the impact of navigator gating parameters on mapping accuracy in a phantom and precision in healthy volunteers.

**Methods:**

The PARMANav sequence (PArametric Radial MApping with Navigator gating) was implemented with 25 single-shot radial gradient-echo acquisitions with five magnetization preparations and lung-liver navigator gating to avoid through-plane motion. Images were reconstructed using region-optimized virtual coils and compressed sensing, followed by model-based registration. An acquisition-specific joint T₁–T₂ dictionary was generated using extended phase-graph simulations. T_1_–T_2_ accuracy was quantified in a phantom and T_1_–T_2_ precision was established in 10 healthy volunteers. Three patients were scanned to demonstrate clinical feasibility.

**Results:**

In the phantom, PARMANav T_1_–T_2_ accuracy was high and insensitive to rejected navigators (< 5% variation for T_1_ and T_2_). In vivo PARMANav T_1_ and T_2_ values were higher than routine values but less variable, both per subject and between subjects: cortex PARMANav T_1_ = 1601 ± 48 ms/T_2_ = 90.8 ± 5.0 ms vs routine T_1_ = 1307 ± 108 ms/T_2_ = 73.3 ± 8.0 ms, medulla PARMANav T_1_ = 2044 ± 82 ms/T_2_ = 90.3 ± 5.4 ms and routine T_1_ = 1560 ± 122 ms/T_2_ = 67.6 ± 5.8 ms. No T_1_ or T_2_ trend was observed for the different NAWW. High-quality maps were obtained in the patients.

**Conclusion:**

With accuracy confirmed in the phantom study and precision demonstrated in volunteers, PARMANav allows for precise and accurate renal joint T_1_–T_2_ mapping during free-breathing while minimizing through-plane motion.

**Supplementary Information:**

The online version contains supplementary material available at https://doi.org/10.1007/s10334-026-01337-8.

## Introduction

In kidney disease, the T_1_ and T_2_ relaxation times reflect underlying changes in tissue composition [1], while a correlation between T_1_ cortico-medullary differentiation (CMD) and renal function has been demonstrated in several renal diseases [2, 3]. T_1_ CMD was proven to be related to the degree of fibrosis, while elevated cortex T_1_ values are strongly associated with poor renal outcome in patients with chronic kidney disease and with allograph kidneys [4] and edema and T_2_ increase with inflammation and edema [5]. Renal T_1_ mapping [1, 5] is typically performed with modified Look-Locker inversion recovery (MOLLI) [6], which is known to underestimate the T_1_ value [7]. Renal T_2_ mapping is less common and is usually performed with a turbo spin-echo sequence, which typically overestimates the T_2_ value.

Instead of mapping a single relaxation time per acquisition, over the past decade, multiparametric mapping techniques have enabled the simultaneous measurement of multiple relaxation times in a single acquisition. These approaches have gained interest thanks to their ability to provide more comprehensive insights into renal structure and function [5], while known pulse sequence imperfections (e.g., slice profile, inversion efficiency) can be incorporated into the fitting model to improve accuracy. Although such techniques have been successfully commercialized for neuroimaging with MR fingerprinting [8], and a large number of variations have been studied for cardiac MRI [9–12], their application to renal imaging is thus far limited to the research setting, partly because they require breath holding, which is not always feasible in kidney disease patients. The development of free-breathing techniques would represent an impactful step forward toward the integration of T_1_ and T_2_ mapping in clinical practice to assess kidney structure in these patients. Recent studies have demonstrated applications for breath-held joint T_1_–T_2_* mapping [13], T_1_–T_2_ mapping [14], and free-breathing T_1_–T_2_* mapping [15] of the kidney at 3 T. Most of the abovementioned techniques require breath holding, which is not always feasible in patients. Free-breathing achieved with respiratory gating [15] relies on the assumption of a constant respiratory cycle duration and uses a two-parameter analytical fit that does not allow modeling precisely the magnetization evolution.

Conversely, many recent multiparametric techniques enable free breathing through the use of state-of-the-art in-plane retrospective motion correction (i.e., registration) between the source images [16–19], but do not account for through-plane motion displacement perpendicular to the image plane and thus invisible to a motion correction algorithm. The kidneys are typically oriented in an oblique pseudo-coronal orientation, with their long axis tilted by about 15–20° relative to the coronal plane [20]. Consequently, respiratory motion of 20 mm produces a through-plane component of 6–7 mm, comparable to the slice thickness in many 2D acquisitions, and can therefore cause a notable slice mismatch despite near-coronal imaging. This effect may alter image intensities in source images since the slice is not consistently excited, for instance yielding apparently increased values in healthy tissues or normal values in a lesion. The reliance on registration, especially non-rigid registration, could also induce additional errors that propagate into the final maps. To achieve free-breathing acquisitions that avoid such discrepancies between its source images, a lung-liver navigator can be used to track the diaphragm position, albeit at the cost of acquisition efficiency. The accuracy of such a technique was previously assessed in a cardiac numerical phantom and reported no dependencies on the number of navigator rejections [21].

In the current study, we aimed to demonstrate that an adaptation of the free-breathing navigator-gated multiparametric mapping technique PARMANav (PArametric Radial MApping with Navigator gating) [21] can be used to obtain accurate and precise parametric maps of the kidney at 3 T while avoiding through-plane motion. To that end, a mapping phantom was used to assess the accuracy of the techniques, while precision was evaluated in healthy volunteers. Clinical feasibility was demonstrated in three patients.

## Methods

### Pulse sequence design

**Fig. 1:**
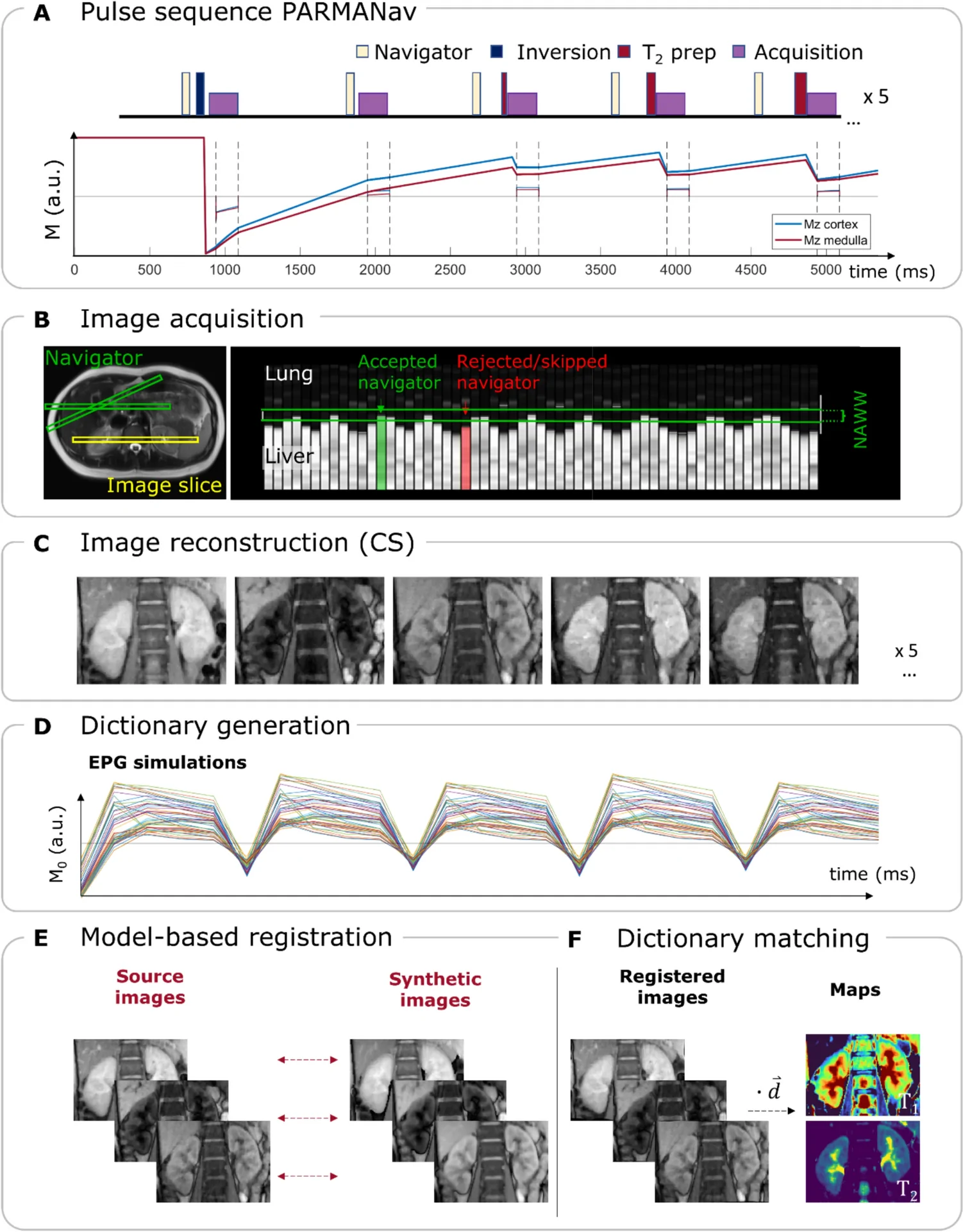
Overview of the free-breathing radial 2D joint T_1_–T_2_ mapping technique PARMANav. **A** Pulse sequence diagram showing the first of five repeated blocks that consist of five differently prepared images, and the simulated magnetization of a healthy renal cortex and medulla. **B** Illustration of navigator (green) and image slice (yellow) placement together with the lung-liver navigator with the acceptance window as two parallel green lines. **C** Example first five source images in a healthy volunteer kidney, reconstructed using compressed sensing (CS). **D** Dictionary generated through EPG simulations across the 25 images. **E** Model-based motion correction aligning source and synthetic images obtained with the dictionary and the first unregistered maps. **F** Final maps are obtained from the pixel-wise dot-product between the registered images and the dictionary ($\mathop{d}\limits^{\rightharpoonup}$)

We adapted PARMANav [21] for renal imaging by implementing a free-breathing acquisition of 25 magnetization-prepared single-shot 2D gradient-recalled echo (GRE) images, each acquired with a continuous golden angle trajectory (Fig. 1). To enable magnetization recovery, the blocks (i.e., a magnetization preparation and a single-shot image acquisition) were repeated every 1 s. To achieve joint T₁–T₂ sensitivity, five different contrast preparations were repeated in series: one adiabatic inversion pulse (a 5.12 ms hyperbolic tangent), no preparation, and three different T₂-preparation modules. This set was repeated five times to ensure contrast diversity throughout the different number of skipped navigators (Fig. 1A, B). The number of repetitions was chosen heuristically.

Respiratory motion was tracked using a lung-liver navigator acquired before each preparation, with slice tracking enabled. To ensure that the timing of all magnetization changes was known, both preparation and readout modules were skipped when navigator rejection occurred.

All data were acquired on a 3T clinical scanner (Magnetom Prisma or PrismaFit, Siemens Healthineers, Forchheim, Germany) with nominal matrix size 192 × 192 (resulting in a 384 × 384 matrix through oversampled radial gridding), 45 continuous golden-angle (68°) radial lines per image (corresponding to 15% radial Nyquist sampling), pixel size = (1.56 mm)^2^, slice thickness 8 mm, flip angle 12°, bandwidth 789 Hz/pixel, repetition time TR 3.49 ms, echo time TE 1.56 ms, acquisition window duration 151 ms, inversion time TI 68 ms (for the image directly after the inversion), and T_2_-preparartion modules echo times 23/45/70 ms. A fixed 5 s delay at the start of the pulse sequence allows for nearly complete magnetization relaxation between successive acquisitions. For all acquisitions we used a 34-channel abdominal-spine coil array.

### Image and map reconstruction

The overall image reconstruction scheme followed our previously described framework [21], combining compressed sensing with total-variation regularization in space and local-low-rank regularization along the contrast dimension [22]**.** The main addition in this work was the integration of region-optimized virtual (ROVir) coils [23, 24] to minimize radial streaking artifacts coming from the arms and the abdominal fat. Briefly, the 192 × 192-pixels central part of the acquired image was automatically selected as the region of interest, and the periphery of the image was designated as the unwanted signal region. A generalized eigenvalue decomposition was used to identify virtual coils that maximize the signal-to-interference ratio (SIR). The smallest set of virtual coils capturing ≥ 90% of the total signal energy was retained.

The compressed sensing regularization weights along the spatial and contrast dimensions were empirically set to ${\lambda }_{S}$ = 0.01 and ${\lambda }_{C}$ = 0.06 to balance undersampling artifact removal and image sharpness (Fig. 1C).

An acquisition-specific signal dictionary was generated via extended phase graph (EPG) simulations in MATLAB (version R2023b, The Mathworks, Natick, Massachusetts, USA), using the exact sequence timing extracted from the raw data to reflect navigator acceptance. Simulations covered a wide range of T₁ values from 0 to 5000 ms in 10 ms increments, and from 5020 to 6000 ms in 20 ms increments, as well as T₂ values from 0 to 450 ms with a 5 ms increment and from 460 to 700 ms with a 10 ms increment, incorporating slice profile effects (discretized in 50 isochromats) and inversion inefficiency correction [25] (Fig. 1D). The magnetization was simulated at the center of each echo and averaged over each radial single-shot echo train, resulting in a 25 × 60 396 (T_2_ > T_1_ cases being excluded) dictionary.

A previously described model-based non-rigid image registration [21] was applied to account for residual in-plane motion that resulted from residual differences in the respiratory phase while accounting for the strong contrast variations in these images (Fig. 1E). In renal parametric mapping, such registration is increasingly used to mitigate small residual displacements between navigator-accepted images and to improve map quality [1, 26, 27]. Here, a first set of maps was generated via pixel-wise dictionary matching without motion correction. Synthetic images with matched contrast and averaged motion were then created and used as references for non-rigid optical-flow-based registration [28]. Final maps were obtained by performing dictionary matching on the motion-corrected complex images (Fig. 1F).

### Phantom study

The ISMRM/NIST phantom [29] (Premium System 130, CaliberMRI, Boulder, USA) was scanned to evaluate the accuracy of PARMANav with ROVir and the kidney-specific dictionary. Single-shot images were separated by intervals of 1 s, which were sporadically extended to 2 s or 3 s such that navigator rejections could be emulated. Clinical routine T_1_ and T_2_ maps (pixel size = (1.4–1.9 mm)^2^, slice thickness 8 mm) were acquired using 5(3)5 MOLLI (inversion time of 156 and 244 ms for the first and the fifth images, respectively) [6], and T_2_-prepared (TE_T2-prep_ = 0;27;55 ms) bSSFP T_2_ mapping [30, 31], respectively. It was compared against the values obtained with gold-standard inversion-recovery spin-echo (for T_1_ relaxation) and spin-echo (for T_2_ relaxation) techniques. The different sequences parameters are reported in Table 1.

**Table 1:** Parameters of the sequences used for T_1_ and T_2_ mapping

	PARMANav	MOLLI	T_2_-prep bSSFP	IR-SE	SE
Applied in	Phantom, HV, Patients	Phantom, HV	Phantom, HV	Phantom	Phantom
Resolution	1.56 × 1.56 mm^2^	1.4 × 1.4 mm^2^	1.9 × 1.9 mm^2^	1.6 × 1.6 mm^2^	1.6 × 1.6 mm^2^
Matrix size	192 × 192	256 × 141	192 × 117	192 × 114	192 × 114
Slice thickness	8 mm			5 mm	5 mm
Flip angle	12°	12°	60°	90°	90°
Bandwidth	789 Hz/pixel	977 Hz/pixel	930 Hz/pixel	130 Hz/pixel	130 Hz/pixel
TR	3.49 ms	2.46 ms	3.03 ms	7000 ms	7000 ms
TE	1.56 ms	1.18 ms	1.04 ms	12 ms	12/25/50/100/ 250/500/ 1000 ms
Preparation modules	Inversion, 3 T_2_-prep (TE = 0;27;55 ms)	2 Inversions (TI = 156;244 ms)	3 T_2_-preps (TE = 0;27;55 ms)	Inversion (TI = 21; 50;100;250;500; 1000;2000;4000 ms)	None
Trajectory	Golden-angle radial	Cartesian			
Acceleration	Radial 6.7x	GRAPPA 2x		None	None
Free-breathing	Yes, with a navigator	Breath-held (13 s)	Breath-held (9 s)	N/A	N/A

The same parameters were used in the phantom and in-vivo scans. HV = healthy volunteers, TI = inversion time

A phantom repeatability study was performed in the ISMRM/NIST phantom. During a first session, PARMANav was acquired twenty times in the T_2_ layer. The phantom was then taken out of and placed back in the scanner for an additional PARMANav acquisition.

### Healthy volunteer study

Ethics approval was obtained from the Ethics Committee of the Canton of Vaud (CER-VD) of Switzerland under reference numbers 2021-00697 and 2022-00934. All participants provided written informed consent to participate prior to enrollment.

PARMANav maps of *N* = 10 healthy volunteers (31.8 ± 9.1 y, 4F) were acquired with four different navigator acceptance window widths (NAWWs of ± 4 mm, ± 8 mm, ± 16 mm, and ± 32 mm) of the lung-liver navigator in a randomized order to study the impact of through-plane motion. Volunteers were instructed to breathe normally. Like in the phantom study, clinical routine breath-held T_1_ and T_2_ maps were acquired using MOLLI and T_2_-prepared bSSFP T_2_ mapping, respectively. 

The T_1_ and T_2_ values of the visible cortex and medulla as well as the CMD ratio (as the ratio T_1cortex_/T_1medulla_) were determined for each map in each volunteer by manually segmenting regions of interest (ROIs) in both kidneys on the T_1_ map. Values from the two kidneys were averaged. The coefficient of variation (CoV) was calculated as the regional standard deviation divided by the mean relaxation time. The acquisition time was recorded for the four NAWWs.

Patient study.

To preliminarily evaluate its clinical feasibility, PARMANav was acquired in three patients (65.7 ± 15.0,1F) as part of an ongoing study on heart failure [32]. Two patients had chronic kidney disease (CKD), one with heart failure with preserved ejection fraction (HFpEF) and one with heart failure with reduced ejection fraction, while one patient had HFpEF but no CKD. In each patient, one map was acquired with a NAWW of ± 8 mm. No routine mapping techniques were acquired due to the constraints of the total MRI protocol duration. Anatomical reference images (T_2_-weighted HASTE) were acquired at the same location for two of the three patients, and at a slightly different orientation for the last patient.

### Statistics

Agreement between the measured and gold-standard values in the phantom was evaluated using the slope and coefficient of determination (*R*^2^) from linear regression analysis for both PARMANav with and without skipped navigator, and with the routine techniques. Bland–Altman analysis was performed to quantify biases and limits of agreement, and a paired t-test was conducted to evaluate statistical significance. For the repeatability study first scan, the T_1_ and T_2_ the mean, SD, CoV, and repeatability coefficient (RC) were calculated for twelve out of the fourteen vials across the twenty measurements (since the last two vials had T_2_ values that were out of the dictionary range). The T_1_ and T_2_ ICC(2,1) coefficients were reported. Bland–Altman analysis of relative difference was performed between the two different scans.

In the healthy volunteers, the T_1_ and T_2_ values of the cortex and medulla were extracted for the four NAWWs and the two routine methods. A Shapiro–Wilk test was performed to assess normality. Their differences, as well as the time of acquisition differences, were tested with a repeated-measures analysis of variance (RM-ANOVA) with post-hoc Tukey analysis. The regional T_1_–T_2_ values and CoV means and standard deviations (SDs) across the healthy volunteers were reported. Bland–Altman analysis was performed to quantify biases and limits of agreement.

## Results

### Phantom study

**Fig. 2:**
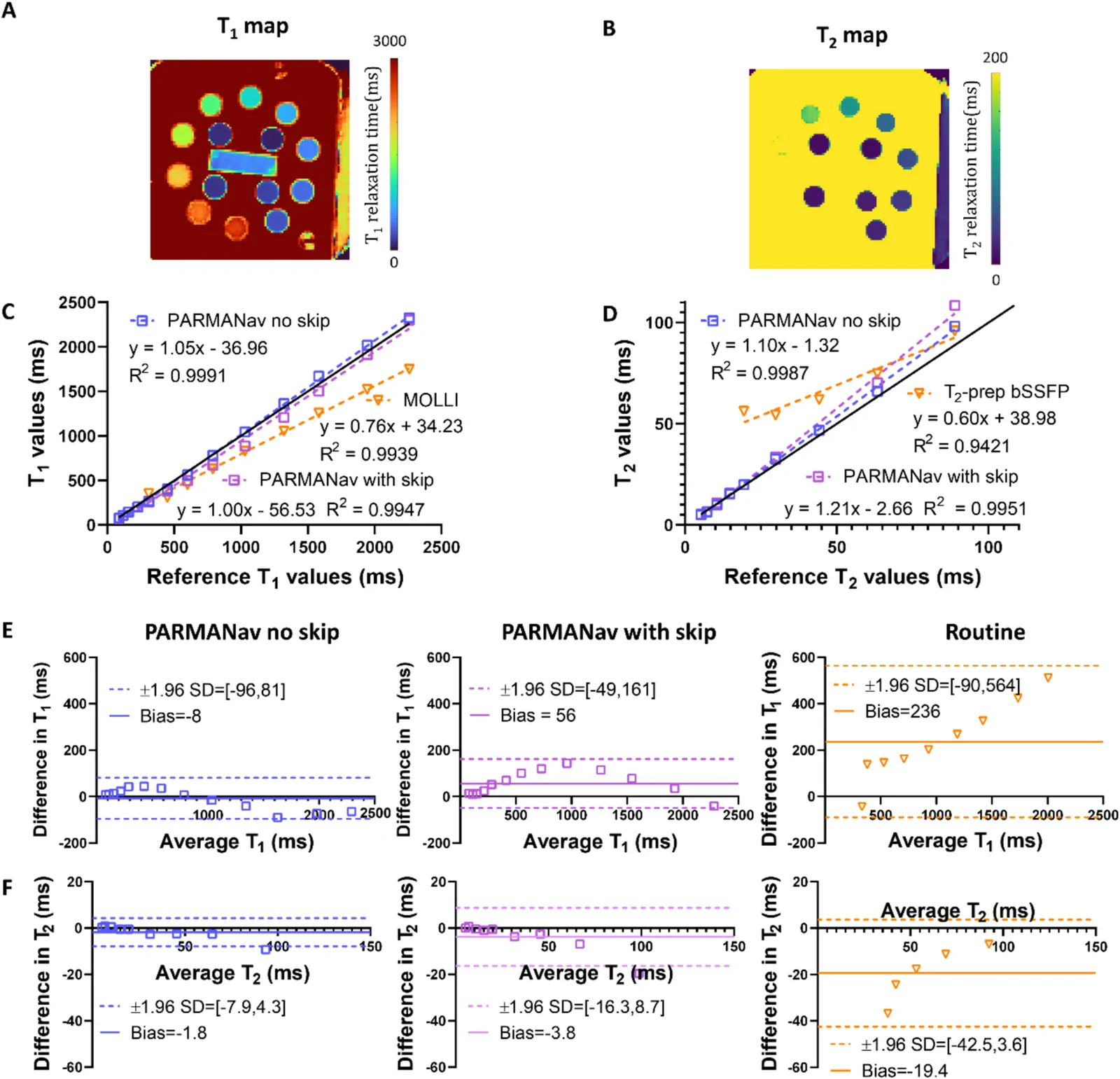
Agreement of PARMANav T_1_ and T_2_ maps in the ISMRM-NIST phantom. **A** T_1_ map of the phantom obtained with PARMANav. **B** T_2_ map of the reference phantom obtained with PARMANav. **C** Linear regression of PARMANav T_1_ values with and without rejected navigator and the clinical routine 5(3)5 MOLLI versus gold standard IR-SE. **D** Linear regressions of PARMANav T_2_ values with and without rejected navigator and the clinical routine T_2_-prep bSSFP in the phantom in the clinically relevant range versus gold standard SE. **E** T_1_ Bland–Altman plot of PARMANav with and without rejected navigator and MOLLI versus gold standard IR-SE. The bias and confidence bounds are reported in the legend. PARMANav presented a larger bias when adding a large number of skips (*N*_skipped_ = 23). **F** Same as **E** for T_2_ with T_2_-prep bSSFP as the clinical routine and SE as the gold standard.

In the phantom, PARMANav showed high agreement with the gold standard, both with and without rejected navigators (*N*_skipped_ = 23) (Fig. 2): the slope of the correlation was closer to identity than the routine technique for T_1_ (1.05 and 1.00 for without and with skipped heartbeat, respectively vs. 0.76 for MOLLI), while there was a larger difference for T_2_ (1.10 and 1.21 vs. 0.60) (Fig. 2C, D). *R*^2^ was above 0.99 for both the PARMANav scans and MOLLI, and slightly different for the T_2_ mapping methods (> 0.99 for the two PARMANav vs. 0.94 for T_2_-prep bSSFP). As with previous studies [21], PARMANav with and without skipped navigators did not significantly differ for T_2_ (*P* > 0.1). A significant difference was reported for T_1_ (*P* = 0.005), with a small average relative difference (6.6%).

Repeatability was high across the 20 phantom scans with an average and maximum vial T_1_ CoV of 0.64% and 2.4%, and an average and maximum vial T_2_ CoV of 1.6% and 6.1%, respectively (Supplemental Table 1). The ICC(2,1) was > 0.99 for both T_1_ and T_2_ relaxation times.

**Fig. 3:**
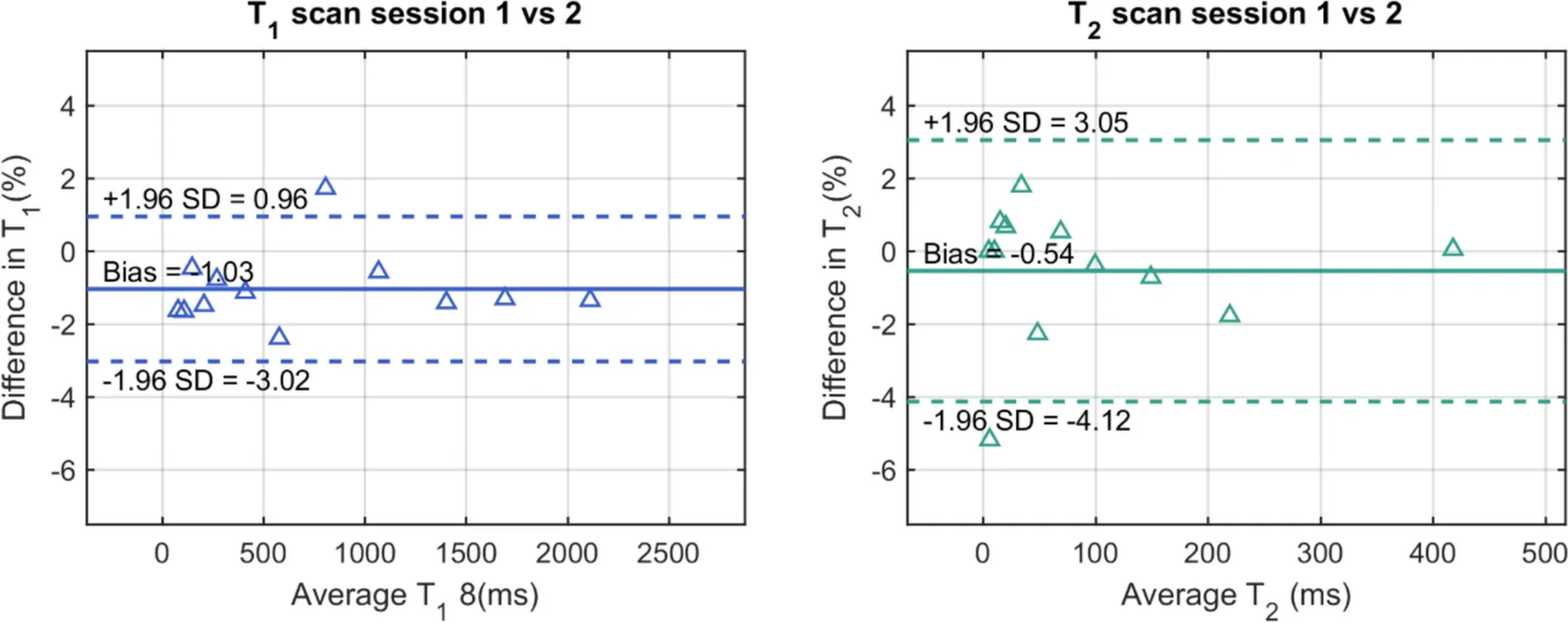
Bland–Altman plot of relative difference between the two scan sessions for T_1_ and T_2_ values. Bias and limits of agreement are reported in the plots

Bland–Altman analyses of the relaxation times obtained in the two scanning sessions showed a bias below 2% for all measurements, with limits of agreement consistent with the values reported in the twenty-measurement experiment (Fig. 3).

### Precision in healthy volunteers

**Fig. 4:**
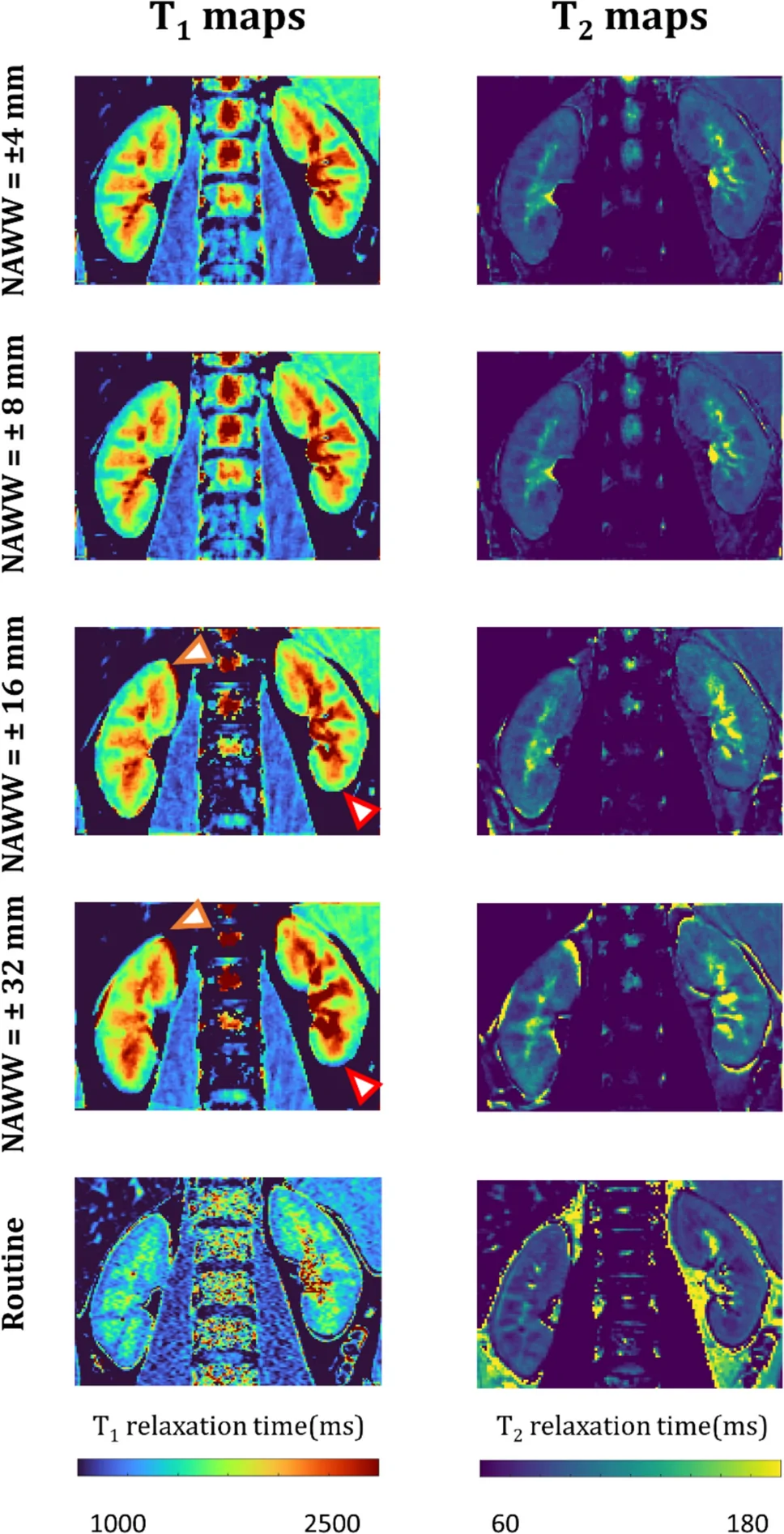
PARMANav T_1_ and T_2_ maps for the different NAWWs compared to the routine techniques. The NAWW did not significantly change the relaxation times in the resulting PARMANav T_1_ or T_2_ maps of the cortex, with the exception of the poles (red arrowheads), where partial volume effect lowered the T_1_ relaxation time. However, the shape and the T_1_ values of the medulla varied with the NAWW (orange arrowhead), likely due to through-plane motion. The PARMANav T_1_ and T_2_ values were consistently higher than those obtained with the routine techniques.

**Fig. 5:**
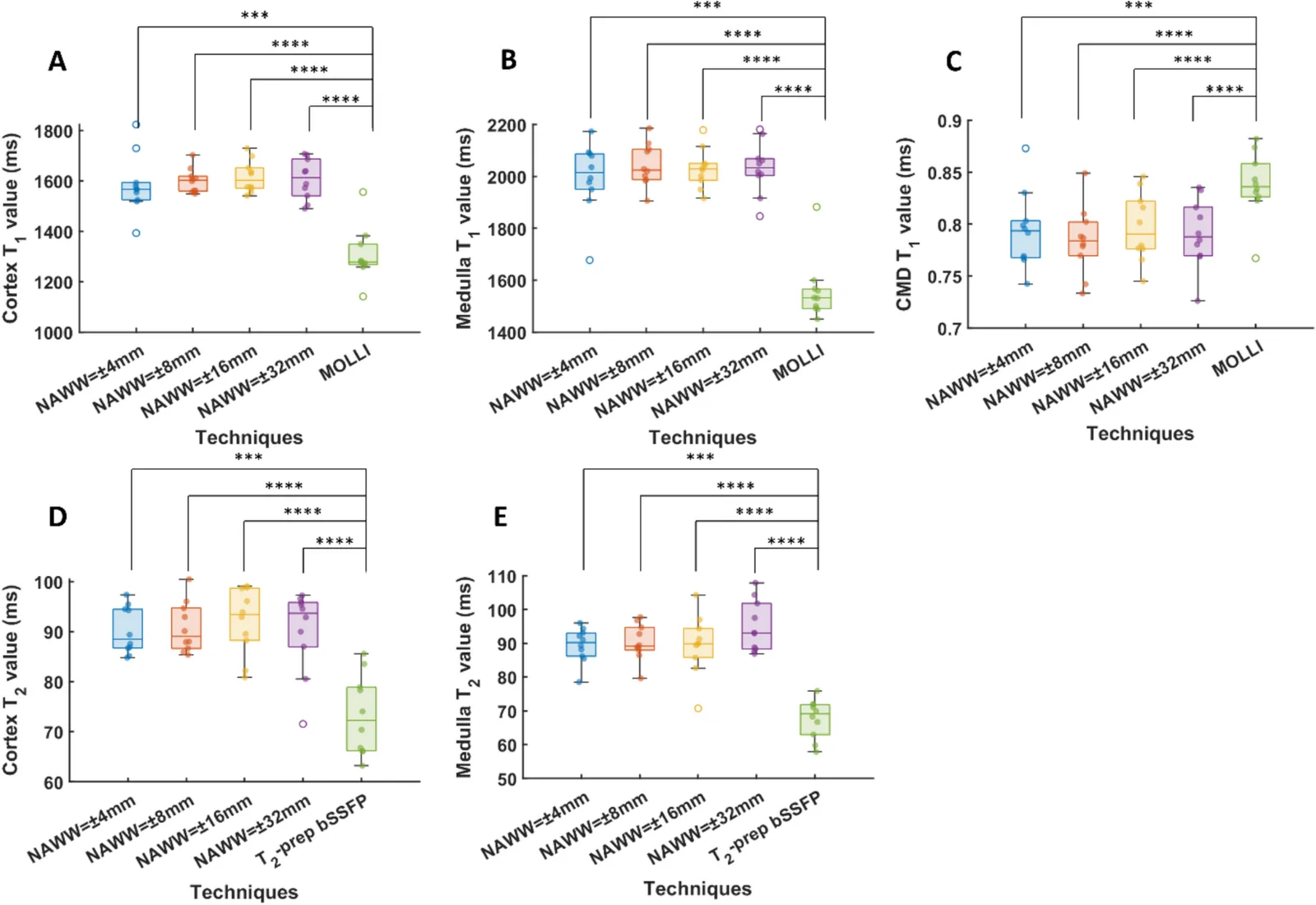
Impact of the navigator acceptance window width (NAWW) in 10 healthy volunteers. **A**–**C** T_1_ values of the cortex and in the medulla and the corresponding CMD ratio for the four NAWWs compared to MOLLI, which is known to underestimate T_1_ values, especially at these higher values. **D**, **E** T_2_ values in the cortex and in the medulla for the four NAWW compared to T_2_-prepared bSSFP

**Fig. 6:**
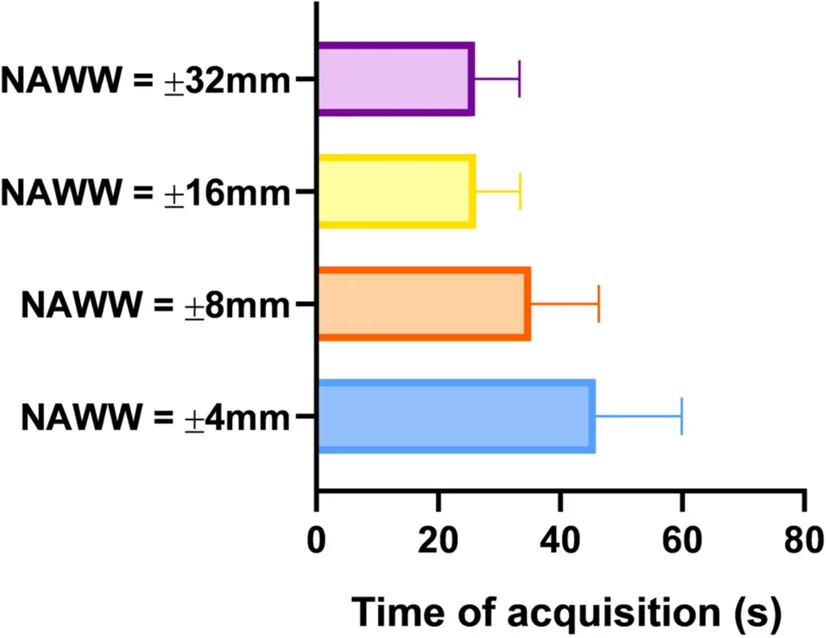
PARMANav acquisition time for the different NAWWs. Acquisition time was longer for smaller NAWW as expected. For NAWW =  ± 16 and 32 mm, the time of acquisition was almost identical, due to no rejection for both NAWWs. The difference was significant between all the times of acquisition (*p* < 0.02) except between NAWW =  ± 16 and 32 mm (*p* = 0.79)

In all 10 healthy volunteers, visually sharp and precise maps of the kidney were obtained with PARMANav (Fig. 4). PARMANav T_1_–T_2_ values and CMD were significantly different from routine techniques, which are known to underestimate T_1_ and overestimate T_2_ [33], but there were no significant differences as a function of the different NAWWs (Fig. 5). The cortex and medulla T_1_ values presented a larger spread for NAWW =  ± 4 mm than for NAWW =  ± 8 mm with more outliers (SD_4mm_ = 117 ms and SD_8mm_ = 48 ms for the cortex). NAWW =  ± 4 mm also resulted in a significantly longer acquisition time (p < 0.02 for NAWW =  ± 4 mm versus all the other NAWWs, Fig. 6). Given these results and the need for balance between low navigator acceptance (at small NAWW) and tolerated through-plane motion (at large NAWWs), NAWW =  ± 8 mm mapping was chosen as the best compromise for further analysis.

PARMANav T_1_–T_2_ values were higher than routine techniques (Table 2). Smaller inter-subject SDs were reported for all relaxation times.

**Table 2:** Cortex and medulla T_1_ and T_2_ mean and standard deviation in ms across the 10 healthy volunteers, as well as the coefficient of variation (CoV)

	PARMANav NAWW = ± 8 mm			Routine	
	Mean ± SD (ms)	CoV (%)		Mean ± SD (ms)	CoV (%)
Cortex T_1_	1601 ± 48		4.1 ± 0.7	1307 ± 108	5.3 ± 2.0
Medulla T_1_	2044 ± 83		4.0 ± 0.7	1434 ± 171	5.4 ± 1.8
Cortex T_2_	90.8 ± 5.0		6.3 ± 2.4	73.3 ± 8.0	4.3 ± 1.3
Medulla T_2_	90.3 ± 5.4		7.4 ± 3.4	67.6 ± 5.8	7.2 ± 5.4

**Fig. 7:**
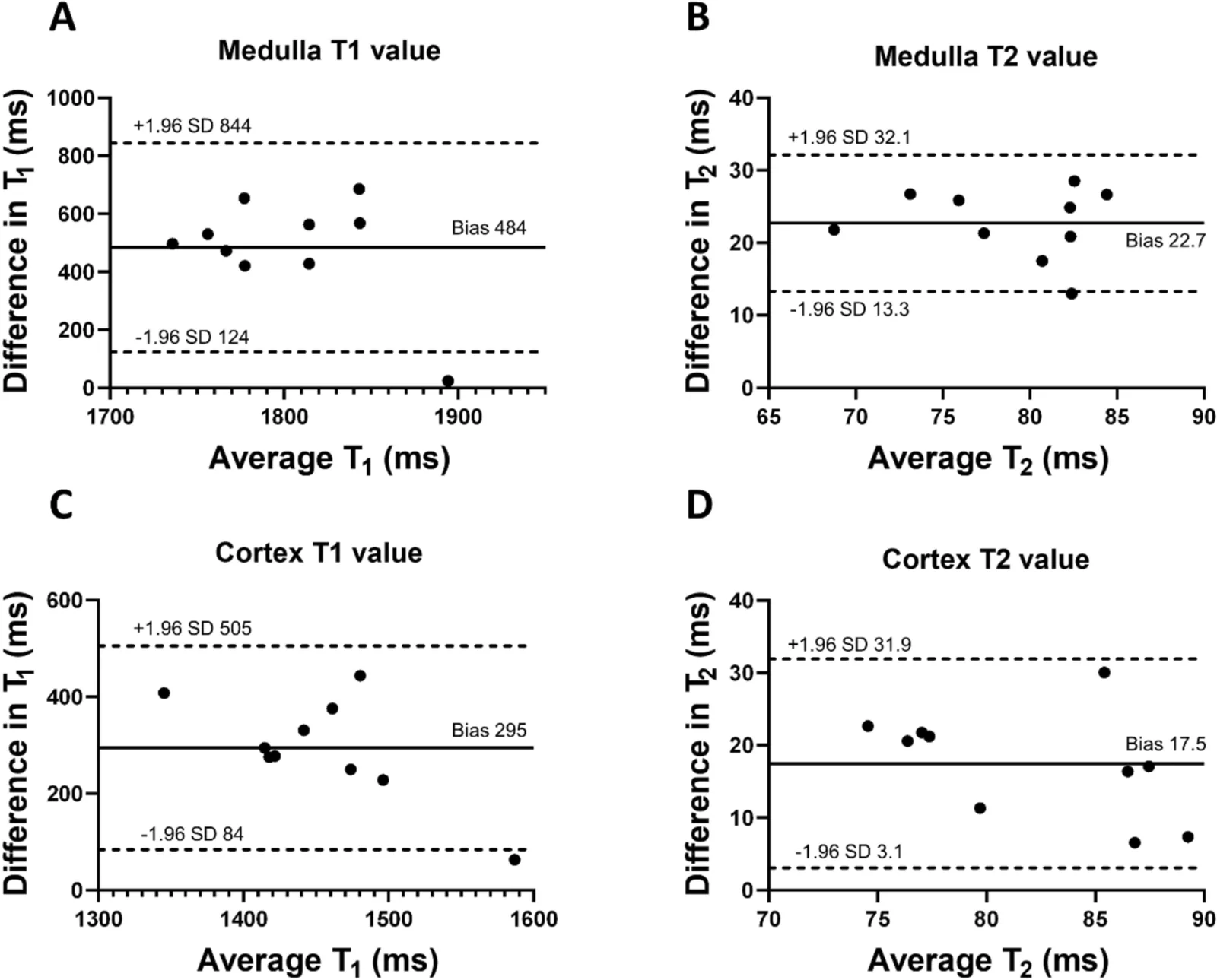
Bland–Altman analyses of the in-vivo agreement between PARMANav with NAWW =  ± 8 mm and the reference techniques. **A** Medulla T_1_ values. **B** Medulla T_2_ values. **C** Cortex T_1_ values. **D** Cortex T_2_ values

The Bland–Altman analysis (Fig. 7) demonstrated a bias against the reference techniques, which is consistent with the phantom results and segmental averages.

### Feasibility in patients

**Fig. 8:**
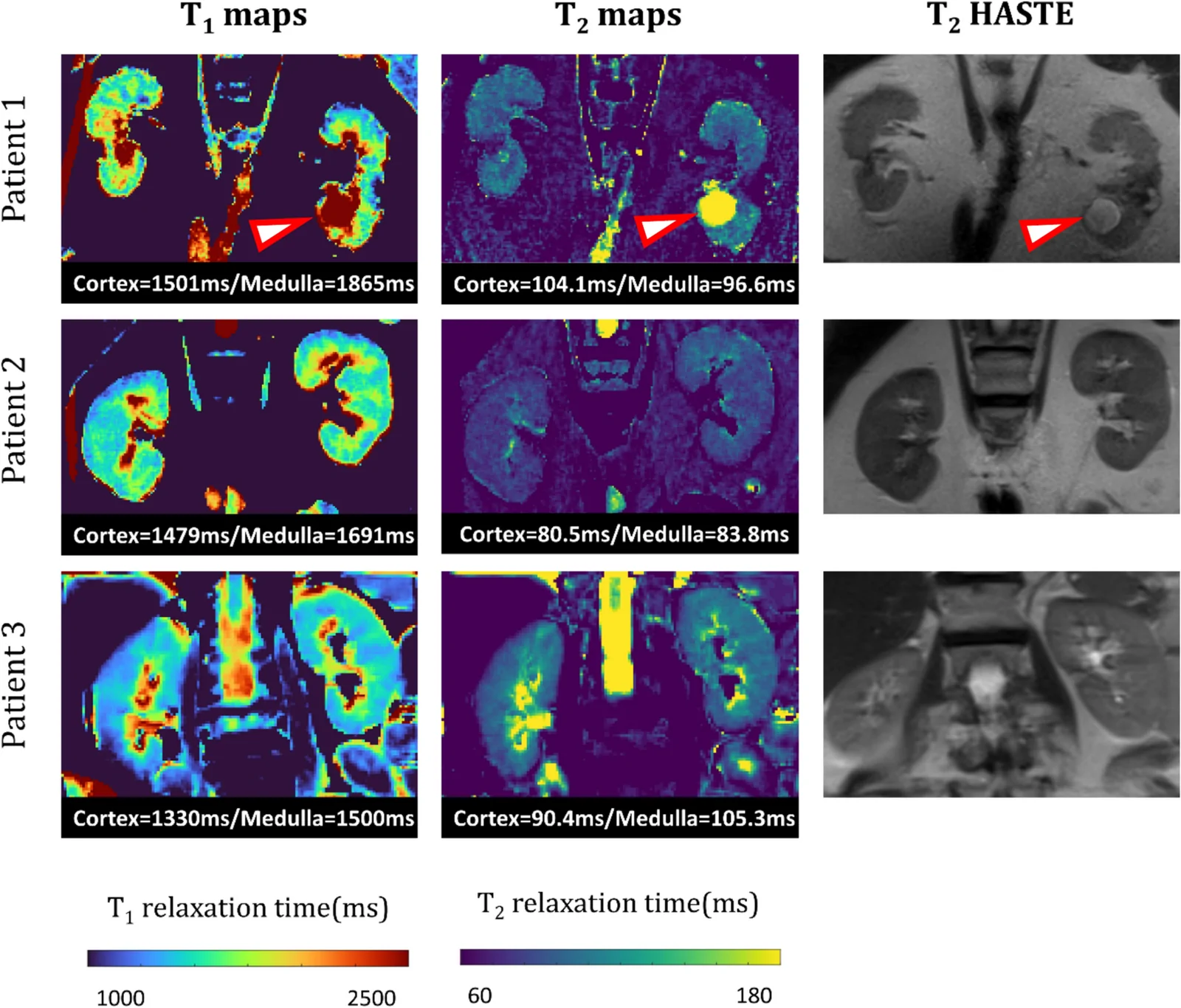
Parametric PARMANav maps obtained in patients. Patient 1 had chronic kidney disease (CKD) and heart failure with preserved ejection fraction (HFpEF) as well as a cyst (red arrowhead). Patients 2 had chronic kidney disease and heart failure with reduced ejection fraction, and Patient 3 had HFpEF without CKD. A T_2_ HASTE anatomical image is provided for anatomical reference

PARMANav was successfully acquired in the three patients and resulted in sharp and artifact-free maps (Fig. 8). A cyst, also visible on the localizer image, could be clearly observed on the maps for Patient 1—the free liquid interior results in very high T_1_ and T_2_ relaxation times. The individual structures in the kidney are more difficult to differentiate in all three patients, while they had a CMD ratio close to one (CMD ratio = 0.80, 0.87, and 0.89 for patients 1, 2, and 3, respectively) as expected [2, 3].

## Discussion

In this work, a free-breathing 2D technique was characterized for joint T_1_–T_2_ mapping of the kidneys at 3T. Previous numerical simulations have shown that the estimated T_1_ and T_2_ values were not impacted by the number of rejected navigators [21]. The mapping phantom accuracy obtained in this study further supports this finding.

The phantom repeatability study confirmed that the techniques are highly stable. When restricting the analysis to the phantom compartments whose relaxation times correspond to renal cortex and medulla values observed in healthy volunteers, the CoV across twenty measurements remained below 3% for T_1_ and below 1% for T_2_. When comparing the different scanning sessions, low bias was observed. With the prospect of deploying this technique across multiple scanners, the repeatability study could be expanded to include a multi-scanner phantom study, similar to recent works of Pasini et al.[34, 35].

PARMANav resulted in precise free-breathing joint T_1_–T_2_ maps of the kidney in the healthy volunteers. It was successfully acquired in all healthy volunteers with all tested NAWWs, which resulted in similar mapped relaxation times. In patients, free-breathing joint T_1_–T_2_ maps resulted in artifact-free maps of high quality with the added advantage that the T_1_ and T_2_ maps are intrinsically registered.

The Bland–Altman study in the healthy volunteers showed a bias compared to routine techniques, which was relatively high for the medulla T_1_ values, but agreed well with the phantom study. T_1_ and T_2_ values were systematically higher for PARMANav, both in the cortex and medulla, with a large limit of agreement.

The higher T_1_ SD for NAWW =  ± 4 mm might indicate that a higher number of navigator rejections impacts the T_1_ precision. To mitigate this effect, a variable flip angle could be introduced for the acquisition, which could also be used to calculate a B_1_ map [11]. Although the impact of the number and timing of rejected navigators (e.g., immediately following the inversion pulse) assessed in a mapping phantom would be valuable, the current experimental setup did not permit such precise control. Future studies using a motion phantom may enable these investigations and facilitate evaluation of accuracy in the presence of motion.

No consistent relationship was observed between NAWW and T_1_–T_2_ precision or accuracy, likely because clear artifact-free borders were avoided during the segmentation process (as illustrated in Fig. 3). This suggests that NAWW selection may be guided primarily by the trade-off between scan duration and through-plane motion tolerance. However, the shape of the cortex and medulla varied with the larger NAWWs, probably due to partial volume effects or limitations in the registration algorithm under conditions of increased motion. The persistence of through-plane motion, even with near-coronal imaging, may explain residual blurring or subtle biases in the quantitative maps. The increased number of outliers observed with NAWW =  ± 4 mm motivated the selection of an ± 8 mm NAWW for subsequent experiments. In addition, parameters such as the flip angle and the number of radial lines per source image could be experimentally optimized and potentially reduced in future studies.

Several breath-held magnetic resonance fingerprinting (MRF) techniques have been proposed for renal mapping, all relying on Bloch equation simulations. Chen et al. developed an abdominal fingerprinting technique for simultaneous T₁ and T₂ mapping with B₁ correction [36]. Compared to this method, which used spiral sampling and Bloch-based dictionary simulation, PARMANav yielded higher T₁ and T₂ values in both the cortex and medulla. More recently, Hermann et al. introduced a breath-held MRF sequence for T₁ and T₂* mapping across four slices [13]. Their reported T₁ values, while still slightly lower, were more closely aligned with those obtained using PARMANav (T_1_ = 1456 ms and 1921 ms in cortex and medulla, respectively, vs. 1601 ms and 2044 ms with PARMANav). MacAskill et al. also presented a breath-held kidney MRF technique for T₁ and T₂ mapping with B₁ correction [14], again reporting shorter relaxation times than those measured using PARMANav. A free-breathing method was proposed by Ding et al. [15] for T_1_ and T_2_* mapping, using respiratory triggering via a respiratory belt. The mapping was based on an analytical equation, which does not allow to model the imperfection of the acquisition (e.g., RF profile, inversion efficiency) and assumes a constant respiratory cycle duration.

The images acquired in patients were sharp and artifact-free. These results are encouraging and suggest that combined T_1_–T_2_ free-breathing mapping with NAWW is possible in CKD patients, whether or not they will suffer from associated heart failure. This should be demonstrated in a future study in a large dedicated cohort, although the presence of heart failure in this failure should not impact the performance of the navigator-gating or PARMANav itself. As a next step, PARMANav should be compared to standard techniques. Future studies should also include a larger number of CKD patients, with different associated comorbidities known to lead to disturbances in breathing such as underlying lung disease or morbid obesity.

This study has several limitations. As mentioned, the number of included patients was small. Furthermore, dictionary-based multiparametric mapping is limited by discretization and computational demands: coarse grids introduce small errors, while finer grids increase generation and matching time. Other limitations of the proposed technique include challenges in sequence optimizations, as navigator rejections may have a greater impact on contrast variability than the parameter being optimized. The navigator only catches superior-inferior motion; although this is typically the largest breathing motion direction, residual through-plane motion in the anterior–posterior direction may remain uncorrected. Due to the complexity of kidney anatomy, only relatively small ROIs can typically be manually segmented. Although a second ROI was drawn on the second kidney to mitigate those effects, automated segmentation of the entire cortex and medulla could provide more representative and robust measurements [37]. Finally, further developments might include semi-automated segmentation, an extension to T_2_* mapping [38] to more sensitively assess oxygenation, and diffusion modules to further characterize fibrosis.

## Conclusion

We demonstrated that the proposed navigator-gated 2D radial GRE sequence PARMANav enables accurate and precise simultaneous T₁–T₂ mapping of the kidneys during free breathing at 3T. The accuracy, as measured in the phantom, was robust to navigator skips, and the NAWW had minimal impact on the accuracy, although very narrow or wide NAWW introduced more outliers. The observed map degradation is potentially due to residual motion and registration errors. Based on these findings, an NAWW of ± 8 mm was selected as a trade-off between scan efficiency and motion robustness.

## Supplementary Information

Below is the link to the electronic supplementary material.

Supplementary file1 (DOCX 14 kb)

## Data Availability

The data that support the findings of this study are available on request from the corresponding author. The data are not publicly available due to privacy and ethical restrictions.
