# Supplementary material for: Navigator-gated free-breathing joint T1–T2 mapping of the kidney
Source: MAGMA. 2026 Mar 5;39(5):751–63. doi: 10.1007/s10334-026-01337-8 (PMC13719845; doi:10.1007/s10334-026-01337-8)
Supplement: Supplementary file 1 [file 10334_2026_1337_MOESM1_ESM.docx]

# Supplemental Material

***Supplemental Table 1. PARMANav T_1_ and T_2_ repeatability observed in the twenty repeated measurements of the ISMRM/NIST phantom.***

| Vials | T_1_ Mean (ms) | T_1_ SD (ms) | T_1_ CoV (%) | T_1_ RC (ms) | T_2_ Mean (ms) | T_2_ SD (ms) | T_2_ CoV (ms) | T_2_ RC (ms) |
| --- | --- | --- | --- | --- | --- | --- | --- | --- |
| 1 | 77 | 0.33 | 0.42 | 0.91 | 5.06 | 0.11 | 2.10 | 0.29 |
| 2 | 107 | 0.47 | 0.44 | 1.31 | 5.60 | 0.23 | 4.20 | 0.65 |
| 3 | 146 | 0.55 | 0.38 | 1.52 | 10.00 | 0.05 | 0.51 | 0.14 |
| 4 | 206 | 0.66 | 0.32 | 1.84 | 15.13 | 0.16 | 1.00 | 0.44 |
| 5 | 268 | 0.63 | 0.23 | 1.76 | 20.04 | 0.07 | 0.35 | 0.19 |
| 6 | 409 | 1.23 | 0.30 | 3.40 | 33.54 | 0.18 | 0.53 | 0.49 |
| 7 | 580 | 1.59 | 0.27 | 4.40 | 48.59 | 0.28 | 0.57 | 0.76 |
| 8 | 797 | 1.97 | 0.25 | 5.47 | 68.13 | 0.35 | 0.52 | 0.97 |
| 9 | 1062 | 3.50 | 0.33 | 9.71 | 99.69 | 0.29 | 0.29 | 0.79 |
| 10 | 1395 | 12.82 | 0.92 | 35.54 | 151.07 | 1.31 | 0.87 | 3.62 |
| 11 | 1671 | 22.96 | 1.37 | 63.65 | 228.09 | 5.34 | 2.30 | 14.80 |
| 12 | 2051 | 51.22 | 2.41 | 137.17 | 446.76 | 27.10 | 6.10 | 75.10 |
